# Bibliometric Analysis of Global Scientific Research on lncRNA: A Swiftly Expanding Trend

**DOI:** 10.1155/2018/7625078

**Published:** 2018-05-27

**Authors:** Xiao Zhai, Jian Zhao, Yiran Wang, Xianzhao Wei, Gengwu Li, Yilin Yang, Ziqiang Chen, Yushu Bai, Qijin Wang, Xiao Chen, Ming Li

**Affiliations:** ^1^Department of Orthopedics, Changhai Hospital, Second Military Medical University, Shanghai, China; ^2^Graduate Management Unit, Changhai Hospital, Second Military Medical University, Shanghai, China; ^3^Department of Oncology, Changhai Hospital, Second Military Medical University, Shanghai, China; ^4^Department of Orthopedics, Panzhihua Central Hospital, Sichuan Province, China; ^5^Department of Endocrinology, Changhai Hospital, Second Military Medical University, Shanghai, China

## Abstract

To investigate trends in long-noncoding (lnc) RNA research systematically, we compared the contribution of publications among different regions, institutions, and authors. Publications on lncRNA were retrieved from Web of Science (WoS) from 1975 to 2017. A total of 3879 papers were identified, and together they were cited 62967 times. The literature on lncRNA had been continuously growing since 2006, and the expansion might continue at a rapid pace until around 2021. China contributed the greatest proportion (63.47%) of lncRNA publications, and the USA ranked second in the number of publications (944 articles), while it had the highest citation frequency (43168 times) and H-index (97). The journal* Oncotarget *has the greatest number of publications on lncRNA research, with 305 papers. The keywords could be stratified into two clusters: cluster 1 (application) and cluster 2 (characteristics). Correspondingly, the “TNM stage,” “epithelial mesenchymal transition (EMT),” “cell apoptosis,” and “overall survival” are research hotspots since 2015. Thus, research on lncRNA showed a swiftly expanding trend, with China making the largest contribution. The focus on lncRNA is gradually shifting from “characteristics” to “application.”

## 1. Introduction

With the technological innovation of RNA sequencing and computational prediction, the past decade has seen a rapid increase in the study of long-noncoding RNAs (lncRNAs). It is estimated that at least 90% of RNAs transcribed by the human genome are lncRNAs [1], and several lncRNA databases have been set up, such as lncRNAdb, NRED, lncRNA Disease, and NONCODE. For example, the NONCODE database (http://www.noncode.org/) lists 233,696 lncRNA transcripts and 144,134 lncRNA genes. lncRNA was previously considered as “junk gene” or transcriptional “noise” [2]; however, it is reported recently that lncRNAs might be associated with many diseases, and the number of lncRNAs with validated functions is growing exponentially. The lncRNA database (http://www.lncrnadb.org) currently lists a conservative 299 functional lncRNA genes.

Several studies have reported that lncRNAs are involved in a cluster of biochemical mechanisms including regulation of gene transcription [3] and methylation [4]. In addition, lncRNAs were reported to play an important role in human diseases such as autism spectrum disorders [5] and thoracic and abdominal aortic aneurysm [6].

Since functional analyses of these lncRNAs are generally more difficult than for coding genes, the majority of functional lncRNAs are still unknown, and no estimation or summary of the relevant scientific output has been demonstrated yet. Based on published books or journal articles, bibliometrics is often employed to assess the tendency of research activity over time, and bibliometric analysis evaluates the literature both quantitatively and qualitatively [7]. Therefore, this kind of statistical analysis can provide bibliographical information on a specific field, issue, institute, or region. Furthermore, this kind of information can assist the government in establishing guidelines, medical consensus, and funding-orientation guidance. Till now, bibliometric analyses have been performed to investigate medical research trends such as gastroenterology [8] and cancer [9].

The proposal of this study is to assess the publication pattern of lncRNA research around the world based on Web of Science (WoS) from 1975 (since WoS includes papers from 1975). This study systematically assessed the publication distribution, stratified by geography, institution, funding agencies, journals, and more. We also assessed the frequency of keywords and then employed bibliometric-mapping tools to demonstrate developments on lncRNAs. Results were analyzed to further understand the structure of this field and to anticipate developments on lncRNA research. Furthermore, this study can provide information for funding agencies to establish related guidelines on lncRNA research.

## 2. Materials and Methods

### 2.1. Sources of the Data and Search Strategy

The data in this article were based on the Science Citation Index-Expanded (SCI-E) of the Web of Science (WoS) from 1975 to 2017, since WoS included papers from 1975. A comprehensive online search was performed on a single day, July 1, 2017, to avoid daily updating bias since the database is still open.

The search key words were referred to MESH terms from PubMed and then were used as follows: TI = (ncRNA*∗* AND long) OR TI = lncRNA*∗* OR TI= lincRNA*∗* OR TI = (linc AND RNA*∗*) OR TI = (RNA*∗* AND long AND (noncoding OR nontranslated OR nontranslated OR noncoding OR nonprotein-coding OR nonprotein coding OR untranslated)) AND Language = English. Regarding manuscript types, only peer-reviewed articles and reviews were included.

Ethical approval was not necessary, since the data were downloaded from the public databases and did not involve any interactions with human or animal subjects.

### 2.2. Data Collection

The txt data download from WoS was imported into Microsoft Excel 2013, GraphPad Prism 5, and VOSviewer. The distribution characteristics such as country, institution, journals, and funding agency were analyzed by WoS and recorded by two authors (Xiao Zhai and Jian Zhao). Bibliometric indicators were extracted from the data, including publication number, citation frequency, and H-index [10, 11]. The data were analyzed both quantitatively and qualitatively.

To adjust for economic condition and population size, statistics on gross domestic product (GDP) and population sizes from the Word Bank and the Central Intelligence Agency for the most recent report were used in the study.

H-index is calculated as a measure of scientific research impact that reflects both the number of publications and the number of citations per publication: a scholar has published h papers, each of which has been cited in other papers at least h times [12].

### 2.3. Statistical Methods

The time trend of the publications was analyzed by fitting mathematic models with GraphPad Prism 5 (GraphPad Software Inc., CA, USA). The logistic growth model, *f*(*x*) = *c*/[1 + *a* × *e* ^(−*b* × (*x* − 1981)^], was used to model the cumulative volume of documentation due to its good fitness and ability to predict future trends in the literature [13, 14]. Symbol *x* represents the year, and *f*(*x*) is the cumulative volume of paper by the year. The time point when the publication growth rate moved from positive to negative is called the inflection point of the logistic growth curve, which is generated by the formula: ”*T* = 1980+⁡ln⁡*a*/*b*”[14]. 

VOSviewer (Leiden University, Leiden, Netherlands) was used to analyze the relations among highly cited references and productive authors. It is commonly used for mapping and clustering of cocitation network analysis. It also clusters citation terms and portrays the key words by color. The density of occurrence of information is portrayed by the size of the circle [15].

## 3. Results

### 3.1. Countries Contributing to Global Publications

**Figure 1:**
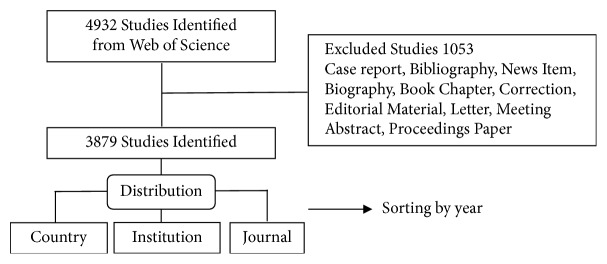
**The inclusion and exclusion process of lncRNA research**.

**Figure 2:**
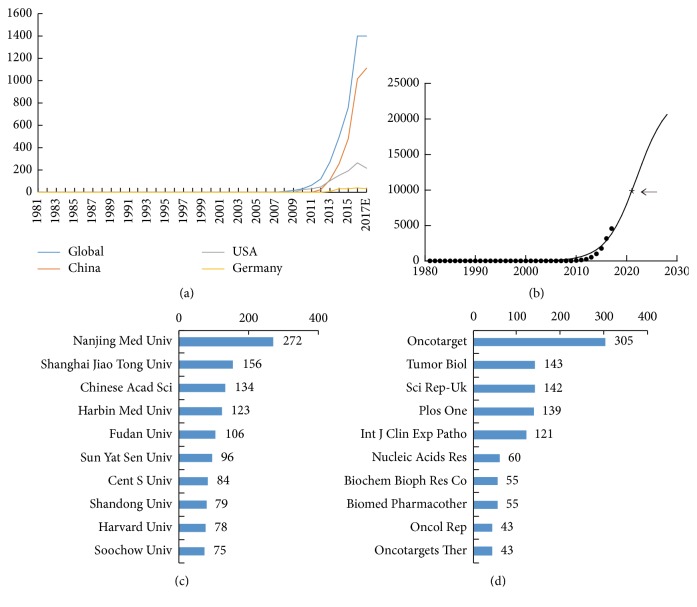
**Contributive characteristics on lncRNA research.** (a) The number of worldwide and the top 3 countries publications on lncRNA research; (b) model fitting curves of growth trends of worldwide publications on lncRNA; (c) the number of publications on lncRNA research from the top 10 contribution institutes; (d) the number of publications of the top 10 popular journals on lncRNA research.

Initially, 4932 papers were retrieved, dating back to 1975. After an exclusion process (Figure 1), 3879 studies were selected for statistical analyses.  Figure 2(a) shows that lncRNAs were continuously reported since 2006. Model fitting curves (*f*(*x*) = 23523.68/[1 + 695518.94 × *e* ^(−0.33 × (*x* − 1981)^]) in Figure 2(b) show a significant correlation between the year and the cumulative number of lncRNA publications. The global inflection point (the time point when the publication growth rate will move from positive to negative) might be the year 2021.

In terms of the most productive countries, China accounted for the highest proportion of published research (2462 papers, 63.47%), followed by the USA (944 papers, 24.34%), and Germany (134 papers, 3.4%). Adjusted by gross domestic product (GDP), China ranked first, with 115.75 articles per trillion GDP. Adjusted by population, Australia came to the fore with 4.52 articles per million population (Table 1).

### 3.2. Citation And H-Index Analysis

According to the analysis of the WoS database, all articles related to lncRNA had been cited a total of 90287 times, an average of 33.8 times per paper. Specifically, the top 100 lncRNA studies (with the highest citation frequency) accounted for 36033 citations (39.91% of 90287) (Supplemental Table 1). In terms of countries, the USA has the most citations (43168) and the highest H-index (97). China ranked second with 30283 citations and an H-index of 76 (Table 1).

### 3.3. Distribution of Institutes Focusing on lncRNA

*Nanjing Med University, China,* had the greatest number of publications with a total of 272 papers, accounting for 1.94% of total published literature in the field, followed by* Shanghai Jiao Tong University, China* (156 publications), and* Chinese Academy of Sciences, China* (134 publications). Among the top 10 productive institutions, nine of them were Chinese institutes, and the other one was an American institute* (Harvard University*), ranking ninth (Figure 2(c)).

### 3.4. Distribution of Published Journals and Funding Agencies Focusing on lncRNA

The journal* Oncotarget *has the greatest number of publications on lncRNA research with 305 papers, followed by* Tumor Biol *(143 papers) and* Sci Rep *(142 papers) (Figure 2(d)). In total, the top 10 journals published 1106 articles, which accounted for 28.51% of all publications in this field.

The top 10 funding bodies are shown in Table 2. A total of 1280 studies (33.00%) were supported by* National Natural Science Foundation of China *(NCSF).* National Institutes of Health *(NIH) ranked second (290, 7.48%). Eight funding agencies from China are shown in the list. In addition, NIH invested in 457 projects with a total funding of $157,041,190 (https://projectreporter.nih.gov/reporter_ChartResults.cfm?icde=36205853), and NCSF invested in 915 projects with a total funding of 377,339,000RMB ($58,052,150) (http://www.letpub.com.cn/index.php?page=grant).

### 3.5. Characteristics of Top 10 lncRNA Articles Cited Most Frequently

In total, the top 10 articles contributed 11386 citations, accounting for 12.61% of citations related to lncRNAs (Table 3). The research by Gupta* et al. *[16] published in 2010 was the most cited (1821 times) paper. Among the 10 most cited articles, two were published in* Cell *[3, 17], two in* Nature *[16, 18], one in* Nature Reviews Genetics *[19], one in* Science *[20], one in* Genome Research *[21], one in* Annual Review of Biochemistry [22],* one in* Molecular Cell *[23], and one in* Genes & Development *[24] (Table 3).

### 3.6. Hotspots of Research on lncRNA

**Figure 3:**
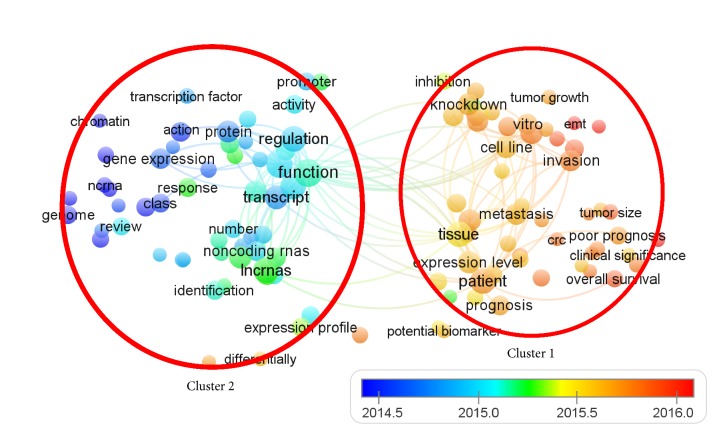
**The analysis of key words.** The mapping on key words of lncRNA; the keywords were divided into two clusters: cluster 1: “application” and cluster 2: “characteristics.” In general, the smaller the distance between two terms, the larger the number of cooccurrences of the terms. A large size of a circle represents that the keyword appears more frequently. The line means that the topics connected on the same line are separated from each other by a comma, a semicolon, or a tab. Based on the average appeared time, key words in blue presented earlier than those in yellow or red. Two terms are defined to cooccur if they both occur on the same line in the corpus file. We set the “100” uppermost appeared lines to be shown.

VOSviewer was used to analyze keywords extracted from the titles and abstracts of 3879 articles included in this study (Supplemental Table 2). As a result, 105 keywords, which appeared more than 100 times, were included and shown in the map. These could be stratified into two clusters: cluster 1 (application) and cluster 2 (characteristics) (Figure 3). High-frequency keywords in cluster 1 were “tissue” (1229 times), “patient” (1142 times), and “progression” (753 times). For the characteristics-related research in cluster 2, the top keywords are comprised of “function” (1632 times), “gene” (1468 times), and “lncRNAs” (1236 times).

In addition, VOSviewer shows different colors according to keyword's average year of appearance, termed “average appearing year (AAY)” (Figure 3). A keyword in blue indicates that its AAY is not recent, and a keyword in red indicates a recent AAY. As a result, “Tumor Node Metastasis (TNM) stage” had the most recent AAY of 2015.98 and appeared 136 times. “Epithelial mesenchymal transition (EMT)” had a second most recent AAY of 2015.96 with 232 appearances, followed by “cell apoptosis” with an AAY of 2015.85 and 171 appearances (Supplemental Table 2).

## 4. Discussion

Bibliometrics and visualized mapping may quantitatively monitor research performance in science and present predictions [25]. Bibliometric study has had impact on other scientific and professional communities. In the case of antimicrobial resistance surveillance, for example, because real-time surveillance data are often unavailable and limited, scholars have used scientometrics and found that it provides a fast, reliable, and global overview of research [26]. As a result, bibliometric studies may be a meaningful reference. In this study, we used the same method as demonstrated in our previous studies [27, 28] and evaluated lncRNA studies with respect to the contributing countries, institutions, journals, and funding agencies.

### 4.1. Global Trends of Research on lncRNA

From 2006 to 2017, a dramatic growth in the global publication number on lncRNA research was validated. The regression growth model of the cumulative amount showed an inflection point in 2021, which means the growth of the publication scale might increase in the coming years. Moreover, there is still the possibility that the increasing trend will go on longer than that expected from the proposed model, because the application of lncRNA as diagnostic biomarkers and as therapeutic agents might arouse more attention.

In terms of country analysis, China published 2642 articles and was the leading country in terms of productivity. Considering the factors of a large Chinese population and GDP, we performed an adjustment and found that China published 115.75 articles per trillion GDP (still ranked no. 1) and 1.79 articles per million population (ranked no. 3). Moreover, we found that although the USA published only 944 articles and was in second place, its total citations and H-index were 43168 and 97, respectively, which surpassed those of the Chinese and suggested that the impact of articles published by the USA might be higher. The quality and creativity of studies from China might be industriously improved in the future.

In terms of journals, we observed that the* Oncotarget *published far more lncRNA research papers, with 305 articles, than other journals. It was indicated that future development within lncRNA would likely be released within* Oncotarget* and the aforementioned journals on the list (Figure 1D).

### 4.2. Research Focuses on lncRNA

The details of the top 10 cited articles are shown in Table 3, and we have listed the top 100 cited articles in Supplemental Table 1. We found that the top 100 studies (2.58% of 3879) had been cited 36033 times (39.91% of 90287), which indicated that these 100 studies might be classic and fundamental for further studies and should be read by those new to the field.

As shown in bibliometric mapping of keywords in Figure 3, it was observed that the focus on lncRNA research is gradually shifting from “characteristics” to “application” over the past 4 years. This is in accordance with the law of the development of a new discipline and translational medicine. Once the basic knowledge of the lncRNA was recognized, its application followed. For application studies, the lncRNA has the potential for diagnosis as a biomarker [29]. It was recommended that lncRNA might be a treatment target in the future. Therefore, further studies should focus on translational research.

In cluster 1 of “application,” the words “function” (1632 times, appearing on average in 2015.14) and “gene” (1468 times, appearing on average in 2015.00) were the most used. The article titled “Long Noncoding RNA HOTAIR Reprograms Chromatin State to Promote Cancer Metastasis” has been cited the most, at 1281 times in total and 227.62 times per year, since this article was published in* Nature* in 2010 [16]. This article proposed that the lncRNA HOTAIR was increased in expression in primary breast tumors and metastases, and the HOTAIR expression level is a powerful predictor of eventual metastasis and death.

Regarding the most recent lncRNA research hotspots, the “TNM stage” [30], “epithelial mesenchymal transition (EMT)” [31], and “cell apoptosis” [32, 33] showed up with the most recent AAY. For instance, in the category “TNM stage,” Cui, Y.* et al. *[30] found that, for those individuals suffering from nonsmall cell lung cancer, higher SNHG1 transcript levels indicated the advanced TNM stage and lymph node metastasis. In the category “epithelial mesenchymal transition”, Hao, Y.* et al. *[31] reported that for prostate cancer the increased transcripts of PlncRNA-1 induced epithelial mesenchymal transition. In the category “cell apoptosis”, Li, Z.* et al. *[32] proposed that in glioma cells the lncRNA MALAT1 promoted proliferation and suppressed apoptosis.

Therefore, we believe that scientific breakthroughs might be related to these hotspots in recent years. As for the prospective application of the VOSviewer map, we suggest that authors could select research topics from the map and demonstrate its importance as frontier hotspot by the map, and funding agents might be suggested to invest in these orientations.

### 4.3. Strengths and Limitations

This bibliometric description and mapping provided a birds-eye view of information on lncRNA-related research for readers to comprehend the history of published lncRNA articles in just a few minutes. In addition, we evaluated the research strength of countries and institutions, which scholars might refer to in order to find cooperative institutions.

During our research using the WoS database, we tried to guarantee comprehension and objectivity. However, we must consider the following limitations. Firstly, only publications written in English were included this study, which, inevitably, missed some significant studies on lncRNA published in other languages. Secondly, other databases such as Scopus or Embase were not analyzed. The WoS database of Science Citation Index Expanded (SCIE) includes publications that represent studies in the discipline, since journals included are selected via a rigorous process under the guidance of the concept of Bradford's law in bibliometrics, and WoS also provides metadata with further distribution refinement. Thirdly, there were still differences between real research conditions and the bibliometric analysis results, since some recently published papers do not have high citation frequency, as reported by Stephan* et al.,* in* Nature *[34]. Lastly, the data in this study are open to expansion, with new studies being published each day, and the increasing trend of publication number might go on for longer than is expected from the proposed model.

## 5. Conclusions 

The literature on lncRNA had been continuously growing since 2006, and expansion is expected until around 2021. China made the largest contribution in the lncRNA research, and* Oncotarget* published the most related articles. All publications can be divided into two clusters, “characteristics” and “application,” and the focus on lncRNA research has been gradually shifting from “characteristics” to “application.” In the relatively new “application” cluster, “TNM stage,” “epithelial mesenchymal transition (EMT),” and “cell apoptosis” may be the latest research hot spots, and related studies may be in the leading position in the lncRNA field in the near future.

## Figures and Tables

**Table 1:** The 10 most productive countries related to lncRNA research.

Country	N	%	N per million population	N per trillion GDP	Total citations	h-index
China	2462	63.47%	1.79	115.75	30283	76
USA	944	24.34%	2.91	50.86	43168	97
Germany	134	3.45%	1.66	33.68	3574	29
Japan	117	3.02%	0.92	23.72	4809	30
England	113	2.91%	1.75	40.53	6250	34
Australia	104	2.68%	4.52	87.47	6642	34
Italy	99	2.55%	1.60	44.57	3930	25
France	72	1.86%	1.08	26.31	3531	24
Spain	66	1.70%	1.36	39.05	3952	23
Canada	62	1.60%	1.75	37.04	1857	18

**Table 2:** Top 10 related funding agencies.

Funding Agency	N	%
National Natural Science Foundation of China	1280	33.00%
National Institutes of Health (NIH)	290	7.48%
China Postdoctoral Science Foundation	64	1.65%
Fundamental Research Funds for the Central Universities	57	1.47%
National Basic Research Program of China	55	1.42%
Natural Science Foundation of Jiangsu Province	55	1.42%
National High Technology Research and Development Program of China 863 Program	36	0.93%
Priority Academic Program Development of Jiangsu Higher Education Institutions	36	0.93%
European Research Council	32	0.82%
Zhejiang Provincial Natural Science Foundation of China	28	0.72%

**Table 3:** Top 10 lncRNA research with the most citation frequency.

Title	First author	Journal	Impact factor	Year	Citations	Citation frequency per year	Main Conclusion
Long non-coding RNA HOTAIR reprograms chromatin state to promote cancer metastasis	Gupta, Rajnish	Nature	40.14	2010	1821	227.62	The lincRNA termed HOTAIR is increased in expression in primary breast tumours and metastases, and HOTAIR expression level in is a powerful predictor of eventual metastasis and death.

Long non-coding RNAs: insights into functions	Mercer, Tim	Nature Reviews Genetics	40.28	2009	1565	173.89	In this review, they focus on the rapidly advancing field of long ncRNAs, describing their conservation, their organization in the genome, their roles in gene regulation and their medical implications.

Evolution and Functions of Long Noncoding RNAs	Ponting, Chris	Cell	30.41	2009	1286	142.89	They reviewed the evolution of lncRNAs and their roles in transcriptional regulation, epigenetic gene regulation, and disease.

Long Noncoding RNA as Modular Scaffold of Histone Modification Complexes	Tsai, Miao-Chih	Science	37.20	2010	1230	153.75	LincRNAs may serve as scaffolds by providing binding surfaces to assemble select histone modification enzymes, thereby specifying the pattern of histone modifications on target genes.

The GENCODE v7 catalog of human long noncoding RNAs: Analysis of their gene structure, evolution, and expression	Derrien, Thomas	Genome Research	11.92	2012	1208	201.33	They presented and analyzed the human lncRNA annotation, produced by the GENCODE consortium within the framework of the ENCODE project and comprising 9277 manually annotated genes producing 14,880 transcripts.

Genome Regulation by Long Noncoding RNAs	Rinn, John	Annual Review of Biochemistry	19.94	2012	1007	167.83	LncRNAs can function as modular scaffolds to specify higher-order organization in RNP complexes and in chromatin states.

Molecular Mechanisms of Long Noncoding RNAs	Wang, Kevin	Molecular Cell	14.71	2011	912	130.29	They discussed the emerging archetypes of molecular functions that lncRNAs execute-as signals, decoys, guides, and scaffolds.

lincRNAs act in the circuitry controlling pluripotency and differentiation	Guttman, Mitchell	Nature	40.14	2011	818	116.86	They performed loss-of-function studies on most lincRNAs expressed in mouse embryonic stem (ES) cells, and found that knockdown of lincRNAs has major consequences on gene expression patterns.

Long Noncoding RNAs with Enhancer-like Function in Human Cells	Orom, Ulf Andersson	Cell	30.41	2010	790	98.75	Depletion of a number of ncRNAs led to decreased expression of their neighboring protein-coding genes, including the master regulator of hematopoiesis, SCL (also called TAL1), Snai1 and Snai2.

Long noncoding RNAs: functional surprises from the RNA world	Wilusz, Jeremy	Genes & Development	9.41	2009	749	83.22	LncRNAs can function via numerous paradigms and are key regulatory molecules in the cell.

## Data Availability

The datasets analysed during the current study are available from the corresponding author on reasonable request.

## Supplementary Materials

Supplementary Materials Supplemental Table 1: details of the top 100 lncRNA articles with the most citations. Supplemental Table 2: details of group items by cluster in VOSviewer.
