# Supplementary material for: Bibliometric Analysis of Global Scientific Research on lncRNA: A Swiftly Expanding Trend
Source: Biomed Res Int. 2018 May 27;2018:7625078. doi: 10.1155/2018/7625078 (PMC5994307; doi:10.1155/2018/7625078)
Supplement: Supplementary Materials — Supplemental Table 1: details of the top 100 lncRNA articles with the most citations. Supplemental Table 2: details of group items by cluster in VOSviewer. [file 7625078.f1.zip › 7625078.f2.docx]

Supplemental Table 2. Details of Group Items by Cluster in VOSviewer

|  | label | Weight  <Occurrences> | cluster | score<Avg. pub. year> |
| --- | --- | --- | --- | --- |
| 1 | function | 1632 | 2 | 2015.144 |
| 2 | gene | 1468 | 2 | 2014.9952 |
| 3 | lncrnas | 1236 | 2 | 2015.2508 |
| 4 | noncoding rna | 1236 | 2 | 2015.0299 |
| 5 | tissue | 1229 | 1 | 2015.4679 |
| 6 | regulation | 1148 | 2 | 2014.98 |
| 7 | patient | 1142 | 1 | 2015.6813 |
| 8 | transcript | 932 | 2 | 2014.838 |
| 9 | progression | 753 | 1 | 2015.6614 |
| 10 | protein | 748 | 2 | 2014.7741 |
| 11 | cell line | 686 | 1 | 2015.5904 |
| 12 | disease | 684 | 2 | 2015.231 |
| 13 | cell proliferation | 674 | 1 | 2015.7359 |
| 14 | expression level | 660 | 1 | 2015.5727 |
| 15 | process | 647 | 2 | 2015.1314 |
| 16 | metastasis | 645 | 1 | 2015.6062 |
| 17 | knockdown | 603 | 1 | 2015.6119 |
| 18 | invasion | 591 | 1 | 2015.736 |
| 19 | proliferation | 578 | 1 | 2015.7007 |
| 20 | mrna | 570 | 2 | 2015.2667 |
| 21 | vitro | 554 | 1 | 2015.6498 |
| 22 | gene expression | 552 | 2 | 2014.7065 |
| 23 | apoptosis | 499 | 1 | 2015.5812 |
| 24 | noncoding rnas | 470 | 2 | 2015.0128 |
| 25 | prognosis | 446 | 1 | 2015.6345 |
| 26 | biological process | 430 | 2 | 2015.2233 |
| 27 | transcription | 420 | 2 | 2014.5738 |
| 28 | migration | 416 | 1 | 2015.8293 |
| 29 | interaction | 413 | 2 | 2015.1889 |
| 30 | activity | 413 | 2 | 2015.0654 |
| 31 | response | 410 | 2 | 2015.2561 |
| 32 | class | 410 | 2 | 2014.622 |
| 33 | expression profile | 396 | 2 | 2015.3359 |
| 34 | present study | 394 | 1 | 2015.6726 |
| 35 | survival | 392 | 1 | 2015.5689 |
| 36 | change | 390 | 2 | 2015.1128 |
| 37 | differentiation | 387 | 2 | 2015.1085 |
| 38 | tumorigenesis | 370 | 1 | 2015.5649 |
| 39 | assay | 361 | 1 | 2015.723 |
| 40 | identification | 361 | 2 | 2015.1302 |
| 41 | genome | 339 | 2 | 2014.3304 |
| 42 | number | 335 | 2 | 2015.0239 |
| 43 | molecule | 317 | 2 | 2014.9716 |
| 44 | poor prognosis | 315 | 1 | 2015.6667 |
| 45 | correlation | 314 | 1 | 2015.5032 |
| 46 | promoter | 302 | 2 | 2014.9371 |
| 47 | microarray | 299 | 2 | 2015.0903 |
| 48 | vivo | 293 | 1 | 2015.6246 |
| 49 | incrnas | 293 | 2 | 2014.8532 |
| 50 | overall survival | 291 | 1 | 2015.8454 |
| 51 | activation | 290 | 2 | 2015.2276 |
| 52 | qrt pcr | 289 | 1 | 2015.7682 |
| 53 | overexpression | 286 | 1 | 2015.493 |
| 54 | understanding | 279 | 2 | 2015.1541 |
| 55 | microrna | 271 | 2 | 2015.0258 |
| 56 | inhibition | 252 | 1 | 2015.377 |
| 57 | sequence | 242 | 2 | 2014.0496 |
| 58 | aim | 239 | 1 | 2015.4644 |
| 59 | mirna | 221 | 2 | 2015.2534 |
| 60 | lung cancer | 216 | 1 | 2015.588 |
| 61 | gastric cancer | 214 | 1 | 2015.5514 |
| 62 | lymph node metastasis | 206 | 1 | 2015.7476 |
| 63 | upregulation | 205 | 1 | 2015.6732 |
| 64 | sirna | 203 | 1 | 2015.2463 |
| 65 | hepatocellular carcinoma | 200 | 1 | 2015.56 |
| 66 | long noncoding rnas | 197 | 2 | 2014.9137 |
| 67 | cell growth | 193 | 1 | 2015.6114 |
| 68 | variety | 193 | 2 | 2014.8446 |
| 69 | tumor tissue | 191 | 1 | 2015.7435 |
| 70 | transcription factor | 187 | 2 | 2014.8663 |
| 71 | oncogene | 185 | 1 | 2015.7568 |
| 72 | tumor size | 184 | 1 | 2015.8207 |
| 73 | cancer progression | 182 | 1 | 2015.5385 |
| 74 | colorectal cancer | 181 | 1 | 2015.7238 |
| 75 | downregulation | 180 | 1 | 2015.5889 |
| 76 | involvement | 180 | 2 | 2014.9556 |
| 77 | hcc | 172 | 1 | 2015.4651 |
| 78 | majority | 172 | 2 | 2014.343 |
| 79 | cell apoptosis | 171 | 1 | 2015.848 |
| 80 | clinical significance | 169 | 1 | 2015.5385 |
| 81 | discovery | 163 | 2 | 2014.7485 |
| 82 | thousand | 161 | 2 | 2014.472 |
| 83 | potential therapeutic target | 152 | 1 | 2015.6711 |
| 84 | tumor growth | 152 | 1 | 2015.6447 |
| 85 | lncrna expression | 152 | 2 | 2015.2105 |
| 86 | cell migration | 149 | 1 | 2015.651 |
| 87 | potential biomarker | 141 | 1 | 2015.5674 |
| 88 | differentially | 138 | 2 | 2015.5652 |
| 89 | nucleotide | 137 | 2 | 2014.9489 |
| 90 | tnm stage | 136 | 1 | 2015.9779 |
| 91 | crc | 136 | 1 | 2015.7647 |
| 92 | human disease | 136 | 2 | 2014.9412 |
| 93 | normal tissue | 134 | 1 | 2015.2687 |
| 94 | adjacent normal tissue | 133 | 1 | 2015.5639 |
| 95 | tumor progression | 131 | 1 | 2015.6107 |
| 96 | action | 126 | 2 | 2014.9286 |
| 97 | quantitative real time pcr | 125 | 1 | 2015.752 |
| 98 | chromatin | 122 | 2 | 2014.2459 |
| 99 | epithelial mesenchymal transition(emt) | 232 | 1 | 2015.9459 |
| 100 | human genome | 120 | 2 | 2014.725 |
| 101 | novel biomarker | 116 | 1 | 2015.4569 |
| 102 | high expression | 115 | 1 | 2015.7304 |
| 103 | gene ontology | 114 | 2 | 2015.6579 |
| 104 | large number | 113 | 2 | 2014.6372 |
| 105 | nsclc | 110 | 1 | 2015.7273 |
